# Harnessing the Prevention Benefits of Antiretroviral Therapy to Address HIV and Tuberculosis

**DOI:** 10.2174/157016211798038551

**Published:** 2011-09

**Authors:** Reuben Granich, Ying-Ru Lo, Amitabh B Suthar, Marco Vitoria, Rachel Baggaley, Carla Makhlouf Obermeyer, Craig McClure, Yves Souteyrand, Jos Perriens, James G Kahn, Rod Bennett, Caoimhe Smyth, Brian Williams, Julio Montaner, Gottfried Hirnschall

**Affiliations:** 1 Department of HIV/AIDS, World Health Organization, Geneva, Switzerland; 2 University of California at San Francisco, San Francisco, USA; 3 Independent Consultant, UK; 4 South African Centre for Epidemiological Modelling and Analysis, Stellenbosch, South Africa; 5 British Columbia Centre for Excellence in HIV/AIDS, Vancouver, Canada

**Keywords:** Cost effectiveness, economics, HAART, highly active antiretroviral therapy, HIV prevention, mathematical models, tuberculosis prevention.

## Abstract

After 30 years we are still struggling to address a devastating HIV pandemic in which over 25 million people have died. In 2010, an estimated 34 million people were living with HIV, around 70% of whom live in sub-Saharan Africa. Furthermore, in 2009 there were an estimated 1.2 million new HIV-associated TB cases, and tuberculosis (TB) accounted for 24% of HIV-related deaths. By the end of 2010, 6.6 million people were taking antiretroviral therapy (ART), around 42% of those in need as defined by the 2010 World Health Organization (WHO) guidelines. Despite this achievement, around 9 million people were eligible and still in need of treatment, and new infections (approximately 2.6 million in 2010 alone) continue to add to the future caseload. This combined with the international fiscal crisis has led to a growing concern regarding weakening of the international commitment to universal access and delivery of the Millennium Development Goals by 2015. The recently launched UNAIDS/WHO *Treatment 2.0* platform calls for accelerated simplification of ART, in line with a public health approach, to achieve and sustain universal access to ART, including maximizing the HIV and TB preventive benefit of ART by treating people earlier, in line with WHO 2010 normative guidance. The potential individual and public health prevention benefits of using treatment in the prevention of HIV and TB enhance the value of the universal access pledge from a life-saving initiative, to a strategic investment aimed at ending the HIV epidemic. This review analyzes the gaps and summarizes the evidence regarding ART in the prevention of HIV and TB.

## INTRODUCTION

Although we have made significant advances in our understanding of Human Immunodeficiency Virus (HIV), after over 30 years we still find ourselves struggling to address an HIV pandemic in which over 25 million people have died [1, 2]. In 2010, an estimated 34 million people were living with HIV, with most living in resource-constrained settings. In these settings the majority of people with HIV do not know that they are infected, many are unable to access antiretroviral therapy (ART), and the occurrence of opportunistic infections such as tuberculosis (TB) is frequent [3-5]. HIV infection is the strongest risk factor for TB and people living with HIV have 20-37 times the risk of developing TB when compared with those not infected [6]. In 2009 there were an estimated 1.2 million new cases of TB among people living with HIV (13% of all TB cases), and TB accounted for 24% of HIV-related deaths [6, 7]. ART is the strongest TB prevention intervention and reduces TB incidence by over 90% in some settings [8-11]. In 2005 and 2009, the G8 group of countries met in Scotland and Italy and committed to achieving Universal Access to HIV prevention, treatment, care and support by 2010 [12]. The need to provide access to ART is now widely accepted and there is a pressing demand for both increased investment and more efficient use of funding in order to achieve and sustain universal access.

Universal access remains a dream for millions of people and faces serious technical, economic and political challenges [13]. There has been an unprecedented investment in confronting the HIV pandemic. The Joint United Nations Programme on HIV/AIDS (UNAIDS) estimates United States (US)$15.9 billion was available for the AIDS response in 2009 and that US$22 billion annually will be needed to meet targets by 2015 [14, 15]. Substantial resource mobilization is ongoing through innovative financing and support strategies including the Global Fund To Fight TB, HIV, and Malaria; the US President’s Emergency Plan for AIDS Relief (PEPFAR); the World Bank and private sector initiatives [16-19]. Despite this unprecedented investment, the HIV pandemic is far from being under control and the challenge is to not only to sustain the response with improved efficiency but to expand efforts to confront the pandemic. The recent global economic crisis continues to impact investment in international and national public health [20, 21]. It has resulted in a significant reduction and re-orientation in public sector investment. This translates into an urgent need to leverage existing domestic health sector and international resources, secure new sources of support, and use existing funding more efficiently. The economic, political and technical challenges contribute to a growing concern that there may be a weakening of the longstanding G8 pledge to achieve universal access and to deliver on the health-related Millennium Development Goals by 2015 [22-24].

### The Prevention and Treatment Gap

The development and use of a public health approach to ART was a significant paradigm shift in our response to the epidemic and has saved millions of lives [25]. Despite considerable scepticism and in some cases open opposition, by the end of 2010 6.6 million people were taking ART, around 42% of those eligible by the 2010 World Health Organization (WHO) recommendations for ART (i.e. those with CD4 cell counts ≤ 350 cells/mm^3^) [4, 5]. However, around 9 million people were estimated to be eligible and still in need of treatment in 2010, with substantial patient attrition along the treatment cascade from HIV testing and staging to ART initiation and continuation [3, 5, 26-28]. Furthermore, the number of new infections continues to add to the future caseload, with around 2.6 million new infections estimated in 2010 alone [3, 5, 29]. Everyone with HIV will eventually need ART to survive, so as many as 28 million people are waiting, mostly without knowing that they are living with HIV, to become ART-eligible before they sicken or die [3, 4]. ART has considerable potential to save lives while reducing HIV transmission [30, 31]; however, without a reduction in HIV incidence it is unlikely that we will be able to meet the UNAIDS target of 15 million on ART by 2015 [13, 32].

We have learned a great deal about what it takes to expand access to treatment in the face of impoverished and resource-starved health systems; however, it remains an enormous challenge to accelerate access to meet the demand [13]. Our current focus is on limiting scarce treatment-dedicated resources to provide life-saving access to ART for people who are markedly immunocompromised. *Treatment 2.0* is focused on increasing access and includes five priority areas: optimising drug regimens, advancing point-of-care and other simplified platforms for diagnosis and monitoring, reducing costs, adapting delivery systems, and mobilising communities. Success of the goals of *Treatment 2.0* is essential to achieve universal access targets and combat the dual epidemic of HIV and TB [33]. However, if we do not significantly reduce HIV incidence to close the prevention gap it is unlikely that we will be able to meet universal access targets including the increasing demand for ART [13]. Scientific evidence supporting ART in prevention has helped to expand the previous public health approach to include serious consideration of the clinical and preventive benefits of treatment for individuals and their communities [25, 30, 31].

### The Human Rights and Community Support Gap

The HIV pandemic highlights the glaring lack of equity and human rights in contemporary global public health. The stark reality that millions of people are at risk for HIV and have no or limited access to HIV prevention, treatment and care represents a significant breach of the fundamental right to health [32-34]. HIV-related stigma and human rights violations further exacerbate the problem [35]. Growing economic disparities in many settings highlight the increasingly inequitable approaches to HIV prevention, care and treatment that exist when comparing rich and poor countries [36, 37]. Engaging and supporting the community as a meaningful partner in the design, implementation and evaluation of HIV programmes is critical for programme success, particularly when the potential for stigma and human rights violations exists [35]. Although many programmes recognize this principle in theory, its practice is challenging and further efforts are needed to implement this essential aspect of a successful HIV/AIDS response. As with any aspect of ensuring high quality HIV services, it is important to provide resources to address the human rights and community support gap when considering the design and implementation of HIV and other health programmes. Early efforts to categorise and cost human rights and community support measures suggest that the costs for these interventions are likely to be dwarfed by spending on other aspects of delivering services [15, 38, 39]. Criminalisation or coercive approaches will not work and HIV programmes will only be successful if they are planned and implemented within a human rights framework that respects people living with HIV.

### The Earlier Access to Treatment Gap

HIV infection is likely a continuum with morbidity and mortality increasing as the immune system declines. Clinical data and feasibility evidence suggest significantly increased risks for individuals with ≤350 CD4 cells/mm^3^ and in 2009 the WHO strongly recommended starting ART earlier for everyone with CD4 counts ≤350 cells/mm^3^ [40]. Although most experts are comfortable with the new ≤350/mm^3^ threshold, data suggest that the majority of people start ART in resource-constrained settings far later than the WHO recommended eligibility criteria [41, 42]. Although access is improving, in sub-Saharan Africa people often start at a median CD4 count of around 100 cells/mm^3^ and mortality remains significantly higher when compared with resource-rich settings [41-43]. Early access to HIV testing and counselling is part of the solution; however, in some settings there may be significant morbidity and mortality after HIV testing while waiting to be eligible for ART. One South African study found that 53% of people die while on the waiting list for ART [44]. One explanation is that patients with higher CD4 cell counts are being monitored too infrequently for the timely start of treatment. There is also evidence to suggest that although mortality rates at higher CD4 levels are lower, they are not zero, and being infected with HIV likely represents a significant impact on morbidity and mortality. In Zimbabwe, a study of postpartum women living with HIV not on ART found a 54 times higher risk of HIV mortality within 24 months postpartum for those with CD4 cell counts less than 200 cells/mm^3^ and 5.4 times higher for CD4 counts 400-600 cells/mm^3^ [45]. The hazard remained elevated at 6.2 times higher for women with greater than 600 CD4 cells/mm^3^ when compared to women who were not infected with HIV [45]. Similarly, North American cohort data showed a 94% increase in mortality for those who started treatment below a 500 cells/mm^3^ CD4 count level when compared to those who started earlier [46]. European and North American cohorts including over 40, 000 patients demonstrated that starting treatment earlier reduced the risk of acquired immune deficiency syndrome (AIDS) or death with those starting before reaching CD4 350 cells/mm^3^ having the most benefit [41]. Other cohort studies also suggest that starting earlier is warranted and most evidence increasingly highlights the negative effects of letting CD4 counts drop too low and the damaging effects of HIV even at higher CD4 counts [47-49].

Although not designed to look at when to start, the *Strategies for Management of Antiretroviral Therapy* (*SMART)* trial sub-analyses and more recent work have suggested that starting earlier was more beneficial, and researchers concluded that HIV may be associated with serious non-AIDS defining events including cardiovascular, renal, and liver disease and non-AIDS malignancies [47, 50-53]. Experience and scientific evidence increasingly suggest that HIV infection is likely a chronic inflammatory disease process, supporting recommendations for an earlier start of ART to suppress viral replication [54-56]. Starting ART earlier, before severe immune compromise, is critical for people living with HIV; however, we still do not know with certainty exactly when to start people on ART after HIV infection. Opinions on starting at CD4 cell counts above 350 cells/mm^3^ or even above 500 cells/mm^3^ remain divided, although some settings are already using 500 cells/mm^3^ or higher. Apart from the subset analysis in the *SMART* and the recently stopped *HIV Prevention Trials Network* (*HPTN) 052* trial [57], there are no data from randomized clinical trials to inform the optimal time to start ART in these patients and guidelines are largely based on evidence from observational studies [58]. Both the *Strategic Timing of Antiretroviral Treatment (START)* [59] and *TEMPRANO* [60] randomized controlled trials are in progress and should provide some additional answers to the question of when to start.

While some programmes rely on clinical staging to determine ART eligibility, most use surrogate markers such as CD4 count to determine an individual’s immune status. However, a single CD4 count only represents a snapshot and everyone living with HIV will eventually need ART to stay alive. The question facing people living with HIV, clinicians and policymakers is how long is reasonable to wait to re-evaluate to determine if a person is immunocompromised enough to be eligible for treatment. Data from 30 international studies and 16 cohorts of untreated adults found relatively low CD4 levels after HIV infection and a fairly rapid progression to CD4 thresholds such as 500, 350 and 200 cells/mm^3^ [61]. The median starting levels and time to eligibility was variable, and in some settings was only a few years after HIV infection [61]. From this perspective and assuming access to ART, decisions whether to start at 200, 350 or 500 cells/mm^3^ may represent only a few months or years earlier in the course of a much longer life span while on ART. From a TB prevention perspective, there is growing evidence that waiting until 350 cells/mm^3^ may be too long [62]. This is an evolving area, and future research on the role of viral load or other immune status monitoring may help us to better tailor our approach to determining the timing of treatment eligibility.

In summary, results of clinical trials and observational cohort studies have now conclusively demonstrated that earlier initiation of ART ≤350 CD4 cells/mm^3^ is warranted, to decrease AIDS-related morbidity and mortality. Furthermore, non-AIDS-related morbidity and mortality has been shown to decrease with even earlier initiation of therapy. Guidelines written for high income countries now recommend starting treatment earlier, before severe immune compromise, and use factors such as CD4 decline, viral replication, and discordant couple status as potential eligibility criteria even at higher CD4 counts [63, 64]. There are potential risks to starting ART earlier, and the downsides of starting earlier in terms of toxicity, resistance and other potential adverse effects require further study. The growing body of scientific evidence supports earlier ART, and in November 2009 the WHO replaced the 200/mm^3^ with 350/mm^3^ as the CD4 cell count threshold for starting ART in resource-limited settings [58]. Additionally, the 2010 ART eligibility criteria included everyone diagnosed with TB.

### The Counselling and Testing Gap

Assuring universal access to prevention, treatment, care and support, regardless of the eligibility criteria for ART, will mean that millions of people with HIV will need to have access to HIV testing and counselling to learn their status. Earlier work in 2006 estimated that 80% of people living with HIV in sub-Saharan Africa did not know their status and 90% were unaware of their partners’ status [65]. In 2007, Kenya, a leader in improving access for HIV counselling and testing, found in a national survey that of those who tested HIV positive, 57% of people eligible for ART by Kenyan CD4 count criteria did not know that they had HIV and a further 28% mistakenly thought they were HIV negative; only 16% actually knew their HIV status [66]. However, 92% of those who knew their status and were eligible were on ART [66]. WHO, recognizing the importance of couples counselling and serodiscordancy, will release new couples counselling guidelines that may include a strong recommendation for providing ART for the HIV-infected partner in a serodiscordant couple.

There has been remarkable success in expanding access to HIV testing and counselling as a result of more countries adopting policies on provider-initiated testing and counselling, and increasing numbers of facilities that provide testing [4, 67]. Despite these efforts, knowledge of HIV status remains inadequate and the estimated median percentage of people living with HIV who know their status is below 40% [13]. Data from countries where recent national population surveys have been conducted show that a median of 12% of women and 7% of men report having had an HIV test in the 12 months preceding the surveys, while the median number of people who report having ever tested is 34% for women and 17% for men [13]. There has been financial investment in expanding HIV testing and counselling, and PEPFAR alone reported providing support for 29 million HIV testing and counselling encounters in 2009 and 86 million over the life of the programme [18, 69]. When combined with the 42% ART coverage estimate, the conclusion is that it will likely require a significant acceleration of the HIV counselling and testing efforts to reach the tens of millions people who are unaware of their status [18]. HIV counselling and testing itself, particularly when it includes couples counselling, can be an effective and cost-effective prevention intervention [69-72]. Community-based efforts outside of health facilities, including home-based couples counselling and testing, have considerable promise to expand access to HIV testing and health care [73-77]. In western Kenya, a private sector company in collaboration with the Ministry of Health, local non-governmental organisations (NGOs), and Centres for Disease Control Kenya was able to test 41, 040 or 80% of the men and women between the ages of 15 and 49 years during a seven-day campaign [73, 75]. In our efforts to improve the basic human right to health through the expansion of HIV services including testing and counselling, ensuring access should be provided within a strong human rights framework that emphasizes the Three C’s: consent, confidentiality and counselling [78, 79]. Our current standard referral approach poses a significant challenge for patients, and access to care and treatment after receiving HIV tests remains problematic in many settings [80, 81]. Expanding HIV testing and counselling efforts cannot be considered successful if access to care including ART is not assured.

### Bridging the Prevention and Treatment Gap

Although there have been some successes, stopping the HIV epidemic requires a re-examination of our current approaches to preventing the transmission of HIV. UNAIDS has recently promoted a “*Prevention Revolution*” [82] that proposes to re-invigorate combination prevention including evidence-based interventions to address behavioural change, ART, other biomedical strategies, and structural, social justice and human rights interventions [83, 84]. While this article focuses on ART as a biomedical prevention intervention, it is clear that single interventions alone are unlikely to be sufficient to control or eliminate HIV and biomedical prevention interventions should be considered as part of a larger effort to optimize combination prevention. It is increasingly evident that prevention efforts are producing results in many countries with generalized epidemics, and in 2010 encouraging declines in HIV prevalence have been reported among young people aged 15–24 years [85]. In some countries reporting data on sexual behaviour as well, this fall in prevalence has taken place alongside increased condom use, increased age at sexual debut and a decrease in the number of young people reporting multiple sexual partners [13]. Clearly, it is not possible to attribute these important downward trends in HIV prevalence to a single prevention intervention; rather, it is likely that a wide range of factors have played a role [13]. These include expanded access to information, education and communication programmes, HIV testing and counselling; condom availability; HIV education in schools and behaviour change interventions; and efforts to reduce stigma and discrimination as well as increased access to treatment. The interaction of these factors has helped shape national policy, societal norms and, increasingly, individual behaviour, with the likely result that fewer young people are becoming infected with HIV [13].

The news regarding progress on developing biomedical interventions is mixed. A recent review reported that of 37 randomized controlled prevention trials reporting on 39 interventions including vaccines, microbicides, and herpes suppression trials to prevent sexual transmission of HIV, only 5 reported a positive effect (defined as intervention significantly reduced the risk of HIV in the intervention arm compared to the control arm) [86]. Only the three male circumcision trials, the Thai vaccine trial and the sexually transmitted infection (STI) study in Mwanza, Tanzania, over a decade ago and of limited generalisability, were effective [86, 87]. Male circumcision clearly has impressive potential in high prevalence settings and access is increasing in many heavily burdened countries [13]. Results released in 2010 from the *Centre for the AIDS Programme of Research in South Africa (CAPRISA) 004* vaginal microbicide trial, using a tenofovir-based vaginal gel, were very promising with an antiretroviral-based microbicide thought to be a few years away from widespread use [88]. Pre-exposure prophylaxis (PrEP) is being assessed in at least 5 ongoing or planned international trials [89-91]. The first results, published in November 2010 from the *Preexposure Prophylaxis Initiative (iPrEx)* study in men who have sex with men, showed a 44% decrease in transmission in those who received a daily drug regimen of tenofovir and emtricitabine [92]. However, data showed that adherence to the medications was a major challenge for participants. More recently, the *FEM-PrEP* trial examining the effects of tenofovir/emtricitabine (Truvada^®^) on HIV acquisition in women in Kenya, South Africa and Tanzania had to be stopped because of futility [93]. Although this cast doubts, two trials announced efficacy at the International AIDS Society meeting in Rome in 2011 and there is renewed confidence in the potential for this intervention; however, it will likely prove difficult to provide scarce antiretrovirals to people living without HIV when many others are dying from lack of access to ART. Additionally, PrEP will also face operational challenges around the need to repeat HIV testing to ensure that only those without HIV receive mono or dual preventive therapy. A vaccine may provide an important future intervention [94, 95]; however, the overall current situation has sparked renewed interest in the potential value of ART in preventing HIV and TB transmission [30, 31].

### Harnessing the Preventive Benefit of Antiretroviral Treatment

The scientific evidence base increasingly supports using ART in the prevention of HIV transmission. This shift is a true game changer, as it enhances the value of the universal access commitment from a critical effort to save millions of lives, to a strategic investment that can also drastically reduce the number of new HIV infections. Prevention efforts focused on people living with HIV make sense from an individual and public health perspective. HIV transmission only occurs from people with HIV, viral load is the greatest risk factor for HIV transmission, and lowering the viral load is critical to interrupting transmission and preventing morbidity and mortality [1, 65, 96]. Sexual transmission of HIV-1 is predicted by viral load and is rare when a person has less than 1500 copies of HIV-1 RNA per mL of plasma [96, 97]. Observational studies have demonstrated the potential of ART in preventing HIV transmission, presumably through its significant effect on lowering the viral load [69, 98]. Couples counselling and ART, as part of a combination prevention intervention study in Uganda, reduced HIV transmission by 98% [69]. The recent 2009 meta-analysis, including 11 cohorts (5021 heterosexual couples), found zero risk of sexual transmission while on ART for a viral load below 400 copies/mL (upper confidence limit of 1.27 per 100 years) [99]. A 2009 randomized controlled study of genital *Herpes simplex* virus treatment among long-term, HIV-serodiscordant heterosexual couples in Africa found a 92% reduction in transmission if the HIV-positive partner was on ART [100]. Although most of the scientific evidence points towards efficacy, a recent study from China using programme data did not find ART effective for preventing HIV transmission [101]; however there were methodological concerns regarding this study’s contradictory conclusions, including the context and lack of data on drug quality, viral load, CD4 or adherence [101-103]. Although there are fears about the potential resumption of risky sexual behaviours while on ART, the proportion of couples who had unprotected sex actually decreased when the HIV-positive partner started treatment [100]. In May 2011, the *HIV Prevention Trials Network (HPTN) 052* trial comparing immediate antiretroviral treatment below 550 CD4 cells/mm^3^ with delayed treatment for the HIV-positive partners in discordant couples was stopped 4 years early due to compelling evidence that immediate treatment reduces HIV transmission in discordant couples by 96% [57].

Further proof of concept that ART interrupts HIV transmission can be found in efforts to prevent mother-to-child transmission [104]. Perinatal AIDS cases have been virtually eliminated in the United States and prevention of mother-to-child transmission has proven to be an important point of departure for other focused prevention efforts [104]. This success is most likely due to the implementation of guidelines for the universal voluntary HIV testing and ART for pregnant women living with HIV for their own health [104]. Unfortunately, in 2008, the majority of the 430, 000 new paediatric HIV infections were in sub-Saharan Africa where there is recent evidence that ART can, under optimal circumstances, be used to decrease transmission to 1% [105-107]. WHO recommends the provision of antiretroviral therapy for all HIV-positive pregnant women with CD4 cell counts ≤350 cells/mm^3^ which, if implemented, could potentially prevent an estimated 75% of mother-to-child transmission of HIV [58, 107, 108]. Although not specifically addressed in the guidelines, it is likely that providing treatment to pregnant women could also have a significant impact on the prevention of sexual transmission of HIV to partners as part of the recently announced drive to eliminate mother-to-child transmission of HIV [109-111].

Analysis of programme data provides growing scientific evidence of the impact of ART on community-level HIV transmission and TB incidence. In British Columbia, Canada a decrease in community plasma HIV RNA levels and a decrease in HIV incidence among injecting drug users was associated with expanded access to ART [112]. Between 2004 and 2008, the numbers of HIV diagnoses in San Francisco fell by 45%, the average viral load among the HIV-positive population decreased by 40%, and the actual HIV incidence fell by one-third between 2006 and 2008 [113]. In Taiwan, a 53% reduction in new HIV cases was associated with free access to ART [114]. Evidence also suggests that expanded access to ART has a significant impact on community-level TB incidence, morbidity and mortality [62, 115]. Some caution is necessary, as some communities have reported increased incidence of HIV among men who have sex with men despite availability of ART [116, 117]. Other studies are in progress and will likely shed additional information on the role of expanded ART coverage on community-level HIV transmission.

### The TB Prevention Gap

Another argument for an earlier start is that ART has a significant role to play in preventing TB morbidity, transmission and mortality [10, 115, 118-122]. Early mortality rates in sub-Saharan Africa are very high: between 8 and 26% of patients die in the first year of antiretroviral treatment, with most deaths occurring in the first few months and TB among the leading causes of death [123]. A recent review concluded that ART reduces the risk of TB by 54-92% [8]. In a randomised clinical trial of 642 patients co-infected with HIV and TB in South Africa, starting ART earlier during TB therapy reduced mortality rates by 56% [47]. A recent meta-analysis reports that ART reduces the individual risk of TB by 67% (95% Confidence Interval [CI] 61 to 73%) [124]. Moreover, ART halves the rate of recurrent TB [125]. Given the devastating impact of TB, we may have to intervene with ART earlier before people living with HIV spend too long in the "TB death zone" which has been defined by some researchers as CD4 <500 cells/mm^3^ [62, 126]. Recognizing this, WHO recently revised its guidelines to recommend ART for all patients with TB irrespective of their CD4 count [58].

Arguably most importantly, two randomised controlled trials were stopped early by their respective Independent Data Safety Monitoring Boards due to significant benefits of earlier ART. In Haiti, patients randomised to start ART with CD4 counts from 200-350 cells/mm^3^ had a two-fold lower risk of TB compared to those who were randomised to defer ART until their CD4 count dropped below 200 cells/ mm^3^ [127]. Most recently, in the *HPTN 052* multi-centre trial featuring study sites in Botswana, Brazil, India, Kenya, Malawi, South Africa, Thailand, the United States, and Zimbabwe, there were 3 cases of TB in those starting ART with CD4 counts between 350 and 550 cells/ mm^3^, and 17 cases in those randomised to defer ART until their CD4 count dropped below 250 cells/ mm^3^ (p-value 0.002) [57]. While the results from *HPTN 052* include a significant benefit for earlier ART for reducing extrapulmonary TB, there was no benefit for pulmonary TB and the results have raised questions regarding the overall value of earlier ART for prevention of TB. Observational studies suggest that ART has been associated with up to a 92% reduction in the incidence of tuberculosis, benefiting both people living with HIV and potentially reducing TB transmission to others [8].

Expansion of ART from 2005 to 2008 has decreased TB notification rates by approximately 202 cases/100, 000 persons/year in South Africa (p < 0.001) [121]. Similarly, expansion of ART from 2005 to 2008 in a community in Malawi has been associated with a 33% (95% CI 27 to 39%) reduction in new cases and a 25% (95% CI 9 to 49%) reduction in recurrent cases. In this community of approximately 0.6 million people, ART expansion averted an estimated 1164 new TB cases and 78 recurrent TB cases [115]. These data support the conclusions of a study modelling the impact of starting to expand ART in 2010 with results determined for the years 2015 and 2050 [10]. In this study, initiating ART two years after HIV seroconversion reduced the incidence of TB by 63%, while delaying ART until 5 years after seroconversion reduced the incidence of TB by 48% at 2015 [10]. Continuing to initiate ART two years after HIV seroconversion reduced the incidence of TB by 95% while continuing to delay ART until 5 years after seroconversion reduced the incidence of TB by 66% at 2050 [10].

### Modelling ART in Prevention of HIV Transmission

Mathematical modelling provides one approach for public health authorities to better understand what we think we know and perhaps most importantly what we need to find out.

Models are perhaps most useful when used to examine the potential impact of public health interventions and to determine programmatic targets for maximal impact. In 2008, WHO scientists, building on previous work by others, used mathematical modelling to focus on a generalized HIV epidemic setting largely driven by heterosexual sex and used data from South Africa, Uganda, Malawi and elsewhere [30, 31, 128-131]. The modelling was inspired by earlier analyses suggesting that rapid scale-up of conventional ART approaches could significantly reduce mortality [132] and have a substantial impact on HIV incidence [30, 129]. The model examines ART for all those with CD4 counts ≤350 cells/mm^3 ^(current WHO recommendations) in a southern African generalized epidemic setting and concludes that, although it would not eliminate HIV, it could save nearly 2.41 million lives over a 40-year time frame; expanding ART to everyone irrespective of CD4 count could further increase the impact, by saving an additional 4.78 million lives, and lead to HIV elimination [31]. Modelling expanded HIV testing and counselling and access to treatment for Washington, DC concluded that the strategy could potentially decrease the number of new HIV infections there by as much as 26% over ten years, and work in San Francisco suggests that incident infections could be reduced by 91% [133, 134]. Another analysis examined the potential of universal testing and treatment for reducing the burden of HIV in sub-Saharan Africa with a focus on linkage to treatment and care [135]. Universal testing and treatment with current levels of linkage to care and loss to follow-up could substantially reduce the HIV death toll and new HIV infections [135]. However, increasing linkage to care and preventing loss to follow-up provides nearly twice the benefits of universal voluntary testing and treatment alone [135]. Scientists in Vancouver have reviewed scientific evidence and modelled data derived from their programme and reached the conclusion that expanding access to ART could markedly reduce HIV incidence, decrease drug resistance, save lives, and be cost-effective [30, 112, 136, 137].

Other mathematical modelling studies have reviewed assumptions and examined ART as prevention [138] in other contexts and have arrived at contrasting conclusions, but a full discussion is beyond the scope of this article [133-135, 139-143]. Models are sensitive to key assumptions and when including a different context or more pessimistic parameters, the results are predictably less optimistic and *vice versa* [139-142]. One modelling group using hypothetical assumptions raised the spectre of widespread resistance [142], but data from programmes providing ART and population-based threshold studies suggest that these assumptions may not reflect the general situation [137, 144-147]. Resistance is of course a serious concern whenever anti-infectious disease agents are deployed and WHO is working with partners to monitor the situation through the WHO/HIVResNet HIV drug resistance (HIVDR) Laboratory Network which currently includes over 30 laboratories covering the WHO's African, South-East Asia, Western Pacific, and Caribbean Regions [148, 149]. Although some transmitted resistance is predictable, with 6.6 million people on treatment and millions more in need there is an imperative to not only maintain but expand access to ART to meet universal access goals while putting drug resistance prevention measures in place. Other authors have raised other issues including the potential importance of adherence, sexual disinhibition, the lack of capacity and overwhelming costs of expanding access to ART [150], as well as the importance of the acute phase of HIV infection [151, 152]. Although modelling is important, programme data, research studies and field trials would need to examine the key thresholds for programme performance raised in the supporting information of the recent *Lancet* paper [31] and in subsequent articles by the modelling community [133, 134, 139-143, 150].

### Economic Impact of ART in Prevention

Although there has been considerable investment in confronting the HIV epidemic, the increasing number of people living with HIV will require expanded access to health services. The epidemic presents a substantial ongoing financial burden, and understanding the economic impact of expanding HIV prevention and treatment services is critical for policy makers, donors, programme managers and the community [15, 153-156]. This is particularly important as some policy makers, economists and health authorities have questioned investment in HIV/AIDS in general and in treatment in particular [157]. Despite the need for better information and improved allocation of resources, the macroeconomic aspects of response to HIV are often not part of the public health discourse and key decisions regarding the impact of public sector spending, deficit reduction and other economic measures are often not considered or left to others to determine [157, 158]. In the past, most resource estimations regarding expanding access to treatment have largely focused on the required increase in resources needed to reach service delivery objectives [155]. Although understanding the costs of increased access to antiretrovirals, laboratory tests and other direct costs is critical for expansion, narrowly focusing on inputs alone can also be characterized as “doomsday costing” as it does not take into consideration the individual, public health and economic benefits of expanding services. With a few exceptions, studies considering the costs of expanding access to HIV services including treatment, when they do focus on the benefits, are primarily focused on the health sector perspective and only rarely focus on the societal perspective. Although difficult to estimate, the positive economic impact of expanding access to ART from the perspective of a societal impact is likely to be substantial. Surprisingly, although billions of dollars have been invested in confronting the HIV/AIDS epidemic, solid information regarding the resource needs and economic impact is relatively limited [156].

There is a critical need for strategic information on the cost of care to evaluate the effectiveness, efficiency, equity, and acceptability of HIV interventions [153, 156]. Although discussing global cost estimations in detail is beyond the scope of this review (and is covered elsewhere in this issue by Kahn *et al*.), more recent “next generation” analyses have suggested that, given the high direct medical costs associated with HIV disease, prevention of new transmission is an important element in limiting economic burden [39, 159]. Consideration of the prevention impact of ART is relatively new but is increasingly being considered as part of WHO/UNAIDS resource estimations for expanding HIV programming to reach universal access targets [15, 39, 160].

In 2010, UNAIDS and WHO launched *Treatment 2.0*, a platform to accelerate the public health approach to ART towards greater effectiveness and efficiency [33]. *Treatment 2.0 *aims to achieve and sustain universal coverage of ART in line with recent WHO normative guidelines to treat earlier (≤350 CD4 cells/mm^3^) to reduce morbidity and mortality and to enhance the potential preventive impact of ART [15, 33]. *Treatment 2.0 *aims to further simplify ART, with a focus on five areas: optimization of drug regimens, access to point of care diagnostics, reduced costs, adaptation of delivery systems and community mobilisation [33].

Historically, cost-effectiveness of HIV prevention and of treatment have been contrasted, to inform the mix of investment in these apparently distinct activities [161]. However, this raised concerns and generated controversy due to fears that treatment for sick individuals would be displaced in favour of more cost-effective prevention [162]. A more recent analysis of future resource needs for AIDS in low- and middle-income countries predicted 20% savings with enhanced targeted prevention and 44% higher costs with broad programme scale-up, but did not consider the HIV infections and costs averted with expanded ART [163]. Other recent analyses have found similar positive results but have not included the prevention impact [164]. Next-generation analyses that include expanding access to ART to those earlier in disease combined with a prevention impact offer an opportunity to align the dual objectives of helping those who are ill while lowering the future societal burden of disease. This relies on traditional costing approaches incorporated into an epidemiologic model that includes the impact of access to ART on HIV incidence and prevalence, health services utilization, clinical progression, and survival [39, 159]. Using this approach, a study from Vancouver incorporated HIV prevention impact and concluded that over 30 years, the highly active ART (HAART) expansion scenario was associated with a net benefit of US$900 million (95% CI US$493 million to US$1.45 billion), and that increasing the HAART treatment rate from 50 to 75% of clinically eligible individuals in British Columbia appeared to be a cost-effective strategy based on this model [159]. These cost-effectiveness results are consistent with public health objectives: all individuals who are eligible for an established life-saving treatment should receive it. Another work examines expanding the offering of ART to HIV-infected individuals with CD4 count ≤ 350 cells/mm^3^ in South Africa and estimated that it would reduce new HIV infections by more than 250, 000 over five years and nearly 1.5 million over 40 years. This strategy would reduce estimated deaths by nearly 3 million, disability adjusted life years by more than US$ 15 million over 40 years, and reduce costs by more than US$3.5 billion over 40 years [39]. UNAIDS and WHO have included prevention in their recent costing of the HIV response [15], and further economic modelling to cost the *Treatment 2.0 *simplification approach to ART is ongoing by UNAIDS and WHO. The favourable findings of economic analyses that include the prevention benefit of ART owes much to the potent combination of high averted inpatient costs, low antiretroviral drug costs, and HIV infections averted; this effect is far greater when ART is part of a combined prevention approach [39, 165].

Economic analysis is helpful to understand the broad implications of a complex intervention like ART in prevention. Integrating diverse data on health care utilization and costs in relation to ART and other interventions allows researchers and policy makers to examine the favourable economics of expanded access to ART. More consistent costing methods and more comprehensive coverage - both by country and level of care - are needed in order for policymakers and other stakeholders to be able to optimally monitor and evaluate the cost and cost-effectiveness of country services for HIV treatment and care [153]. Additional economic modelling that allows for modification of parameter value and incorporates the prevention impact of expanding HIV services including ART will be invaluable to increase the robustness and subtlety of our understanding of ART in prevention.

### Ongoing Scientific Evaluation

As part of the ongoing expansion of ART to meet universal access targets, there is a need for further scientific evaluation and discussion regarding public health decision making on how to best use ART in the prevention and control of HIV/AIDS [30, 31, 138, 166]. In November 2009, WHO held two meetings to convene stakeholders for a discussion of ART in prevention designed to explore ethical and human rights issues, further define and clarify research priorities, and review acceptability and feasibility issues. WHO and others are engaged in further modelling on the impact of ART on TB, the relative importance of drug resistance and other assumptions, the effect of combination PrEP and ‘test and treat’, effects on prevention of mother-to-child transmission, an in-depth economic analysis of the various strategies, and a systematic comparison of different models of providing testing and counselling, and of the ethical practices around testing and counselling. There are a number of planned field trials and analyses including ongoing and planned work in Vancouver, British Columbia [167], Washington, District of Columbia (DC) and the Bronx in New York City [168, 169], San Francisco, California [170], Botswana [171], and KwaZulu Natal, South Africa [172]. In May 2011, the British Columbia Centre for Excellence in HIV/AIDS co-hosted a meeting on ART in prevention with WHO, UNAIDS, the International AIDS Society, the National Institutes of Health and others [173]. It reviewed research related to the prevention of HIV and TB. Participants discussed research priorities including planned research projects (see article in this issue for a detailed description of the projects). Scientific and community opinion leaders have called for expansion of access to treatment and ongoing research on ART in prevention [30, 39, 172]. Funding opportunities are increasing and more data on this important topic will be made available in the near future as our experience and evidence base regarding expanding access to ART increases [30, 138, 172].

### Time for a New Treatment Paradigm?

A new paradigm is needed that includes treatment as part of the solution to ending the epidemic and that maximizes conventional prevention interventions. Our current response to HIV is often fragmented and unnecessarily complicated, which translates into a lack of effectiveness and increased costs for both programmes and patients. Prevention, treatment, care and social support programmes could be better integrated in order to effectively use scarce resources. A new approach needs to include a redoubling of efforts to deliver evidence-based, tailored interventions including individual and community-focused prevention services that include affordable, simplified drugs and diagnostics focused on improving access to HIV care for the majority of people living with HIV. Two key opportunities now present themselves that have the potential to hasten and expand the twin goals of savings lives and preventing new HIV infections. Firstly, the ongoing efforts to simplify current ART through simplified antiretroviral regimens will render treatment easier to administer and take and have longer lasting impact for individuals and public health programmes. Secondly, it is also increasingly clear that universal access to ART will have a significant impact on HIV and TB transmission. When combined, expansion of access to ART using simpler, more effective approaches and the use of ART as part of combination prevention will be critical in reaching the goals of universal access and will likely result in dramatic cost savings over the medium and long term.

Our challenge is to understand how best to use new information regarding the role of ART for a reinvigorated, more effective and sustainable global response to AIDS. The therapeutic concepts regarding earlier treatment are not necessarily novel as many researchers, clinicians, programme managers and patients have been calling for earlier access – what *is* potentially new is a focus on simplification with accelerated expansion and full integration of treatment as a key aspect of HIV prevention efforts.

## CONCLUSION

HIV is an infectious disease that, with the right interventions delivered within a human rights framework, can be controlled and possibly even eliminated. UNAIDS and WHO have called for 15 million people to be on ART by 2015 [15] and if we do not achieve universal access to HIV services, millions of people will die before accessing ART. ART has considerable benefit both as treatment and in preventing ongoing HIV and TB transmission, and it is likely that it will be increasingly considered a key element of combination prevention and as part of the solution to ending the HIV epidemic.

## ABBREVIATIONS

AIDS: = Acquired Immune Deficiency Syndrome
ART: = Antiretroviral Therapy
CAPRISA: = Centre for the AIDS Programme of Research in South Africa
CI: = Confidence Interval
DC: = District of Columbia
HAART: = Highly Active Antiretroviral Therapy
HIV: = Human Immunodeficiency Virus
HIVDR: = HIV Drug Resistance
HPTN: = HIV Prevention Trials Network
iPrEx: = Pre-exposure Prophylaxis Initiative
PEPFAR: = United States President’s Emergency Plan for AIDS Relief
PrEP: = Pre-exposure Prophylaxis
SMART: = Strategies for Management of Antiretroviral Therapy
START: = Strategic Timing of Antiretroviral Treatment
TB: = Tuberculosis
UNAIDS: = Joint United Nations Programme on HIV/AIDS
US(A): = United States (of America)
WHO: = World Health Organization
